# Supplementary figures and images for: Low-intensity pulsed ultrasound therapy suppresses coronary adventitial inflammatory changes and hyperconstricting responses after coronary stent implantation in pigs in vivo
Source: PLoS One. 2021 Sep 13;16(9):e0257175. doi: 10.1371/journal.pone.0257175 (PMC8437271; doi:10.1371/journal.pone.0257175)

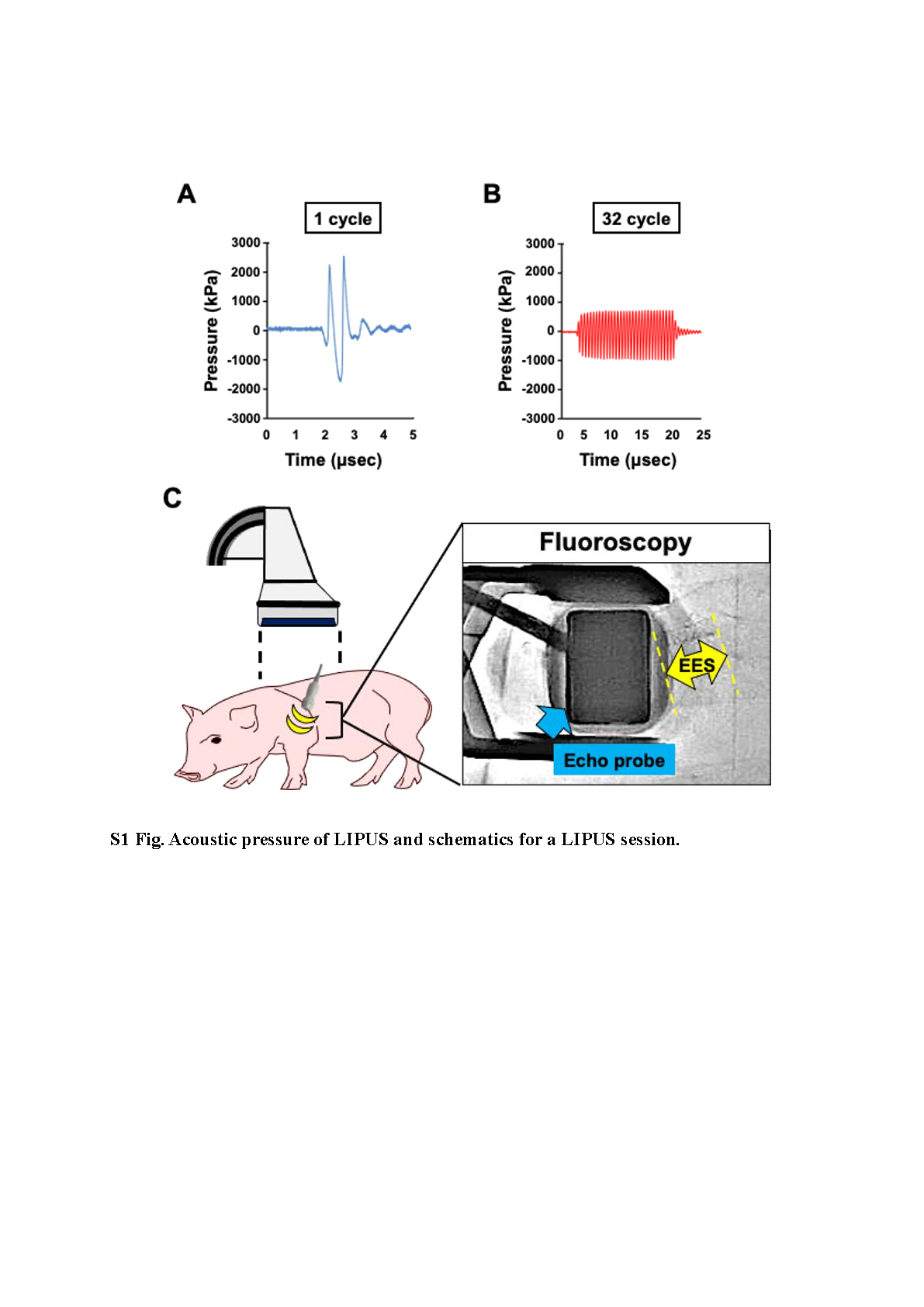

Supplement: S1 Fig — (A) Acoustic pressure at 1 cycle which is chosen for clinically available diagnostic ultrasound devices, and (B) at 32 cycles for the present study. (C) Schematics explaining that 3 sites for one LIPUS session, including the sites proximal and distal to the stents, middle portions of the stents. Target segments were determined by co-registering radiopaque echo probe with the stent architecture. LIPUS = low-intensity pulsed ultrasound. (TIFF) [file pone.0257175.s005.tiff]

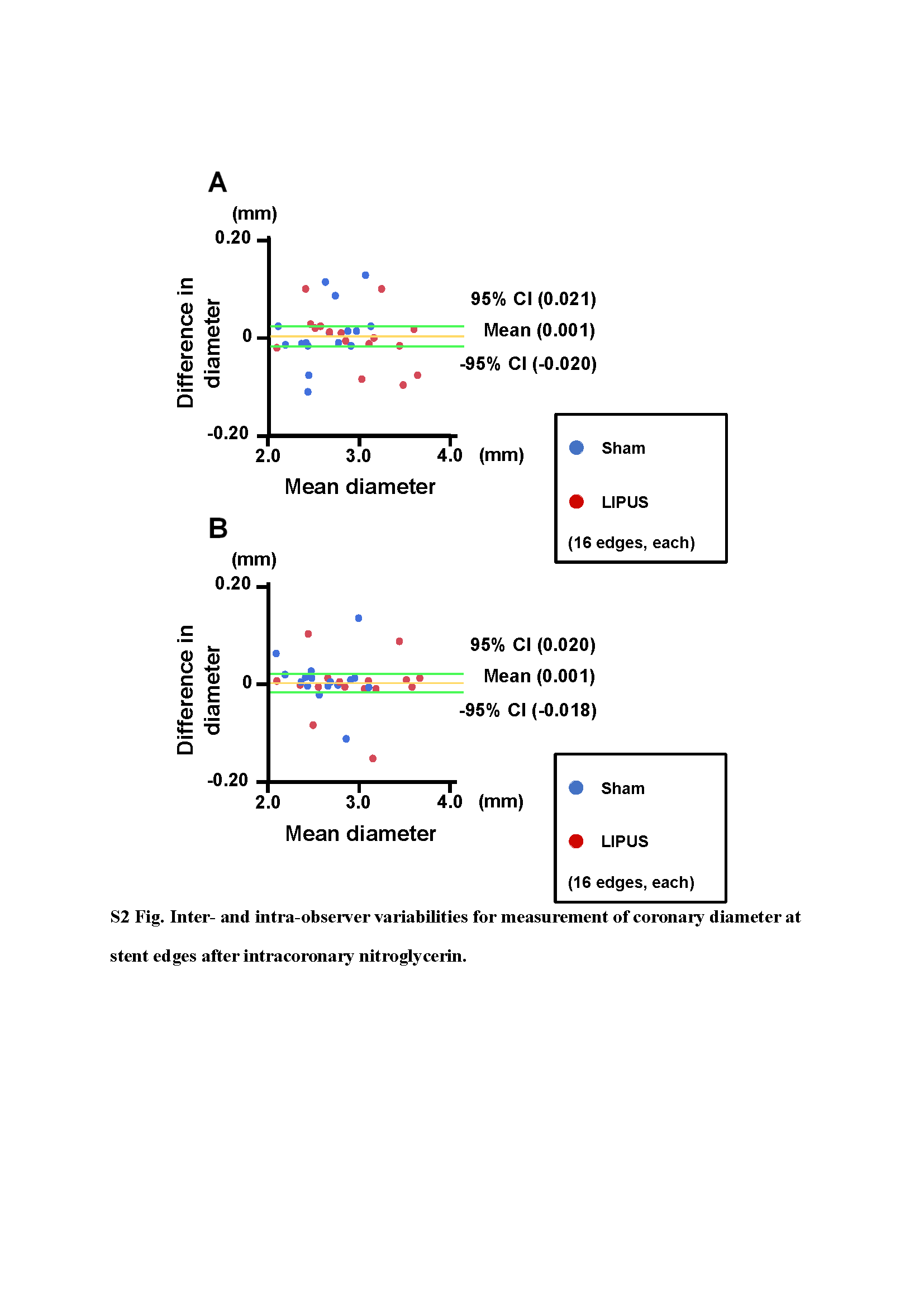

Supplement: S2 Fig — (A) Interobserver variability. (B) Intraobserver variability. Bland-Altman plots of differences in average coronary diameter 5 mm away from stent edges after intracoronary nitroglycerin (10 μg/kg) are shown. Green lines show 95% CI; yellow lines, mean. (TIFF) [file pone.0257175.s006.tiff]

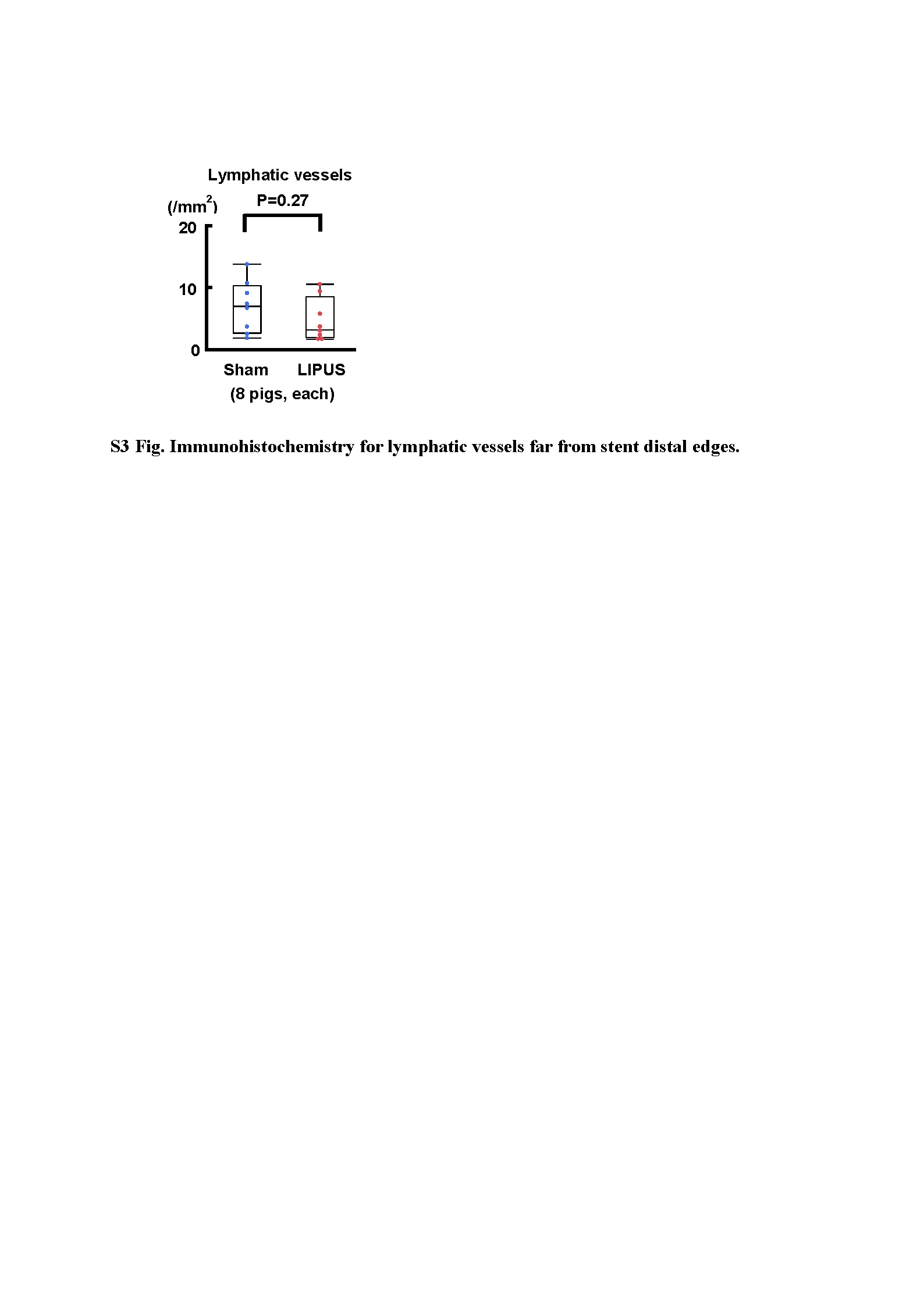

Supplement: S3 Fig — (TIFF) [file pone.0257175.s007.tiff]

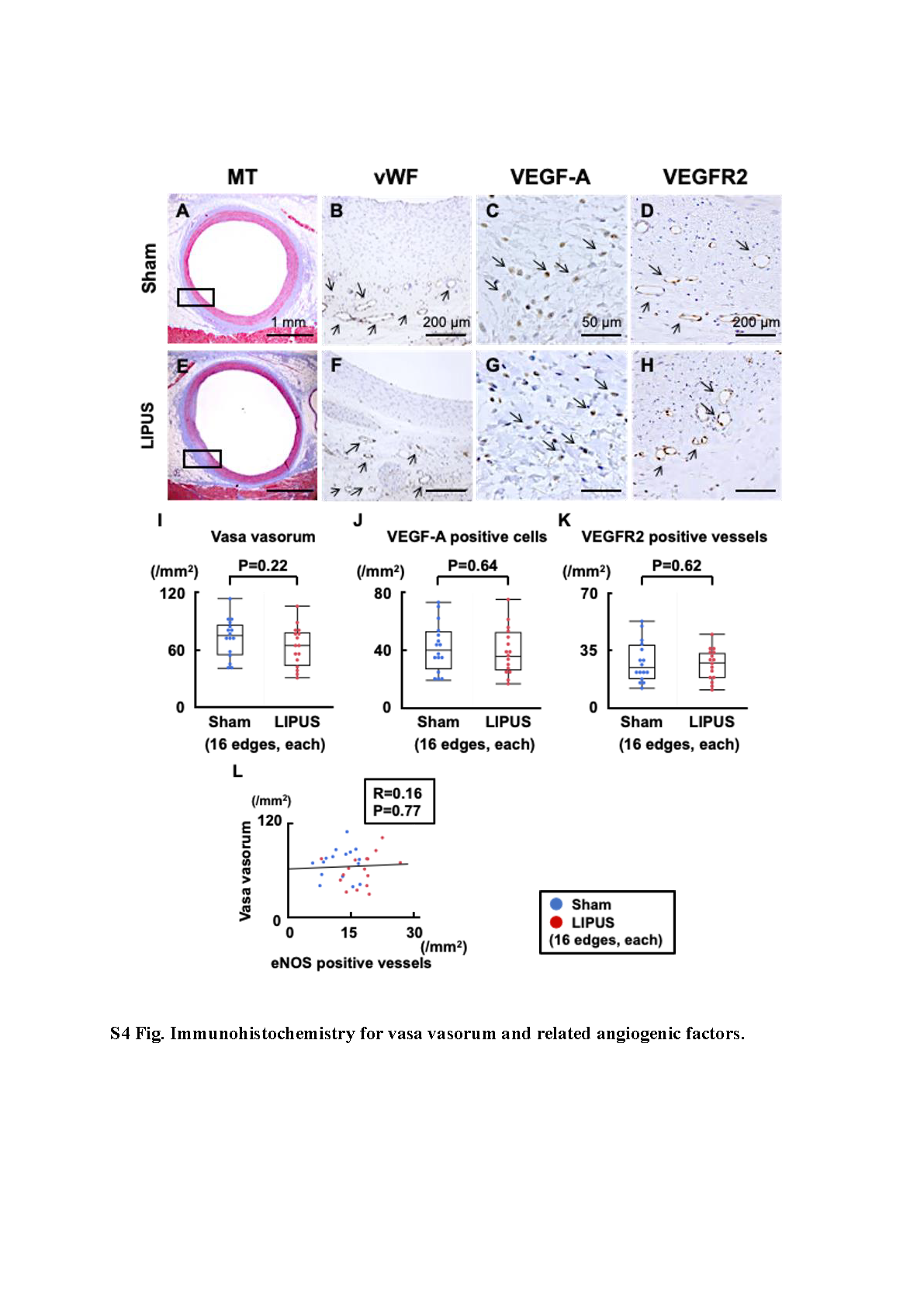

Supplement: S4 Fig — (A and E) Low-magnified MT stainings, and representative immunohistology of (B and E) vWF-positive vasa vasorum, (C and G) VEGF-A-positive cells, and (D and H) VEGFR2-positive cells in the adventitia of vasoconstricting portions. As denoted by arrows in (B through D and F through H), cellular structures positive for (I) vWF, (J) VEGF-A, and (K) VEGFR2 were significantly increased in the LIPUS group as compared with the sham group. (L) No correlation between vasa vasorum vs. eNOS-positive cells. Results are expressed as mean±SEM. eNOS = endothelial nitric oxide synthase; MT = Masson’s trichrome; SEM = standard error of mean; VEGF = vascular endothelial growth factor; VEGFR = vascular endothelial growth factor receptor; vWF = von Willebrand factor; other abbreviations as in S1 Fig. (TIFF) [file pone.0257175.s008.tiff]

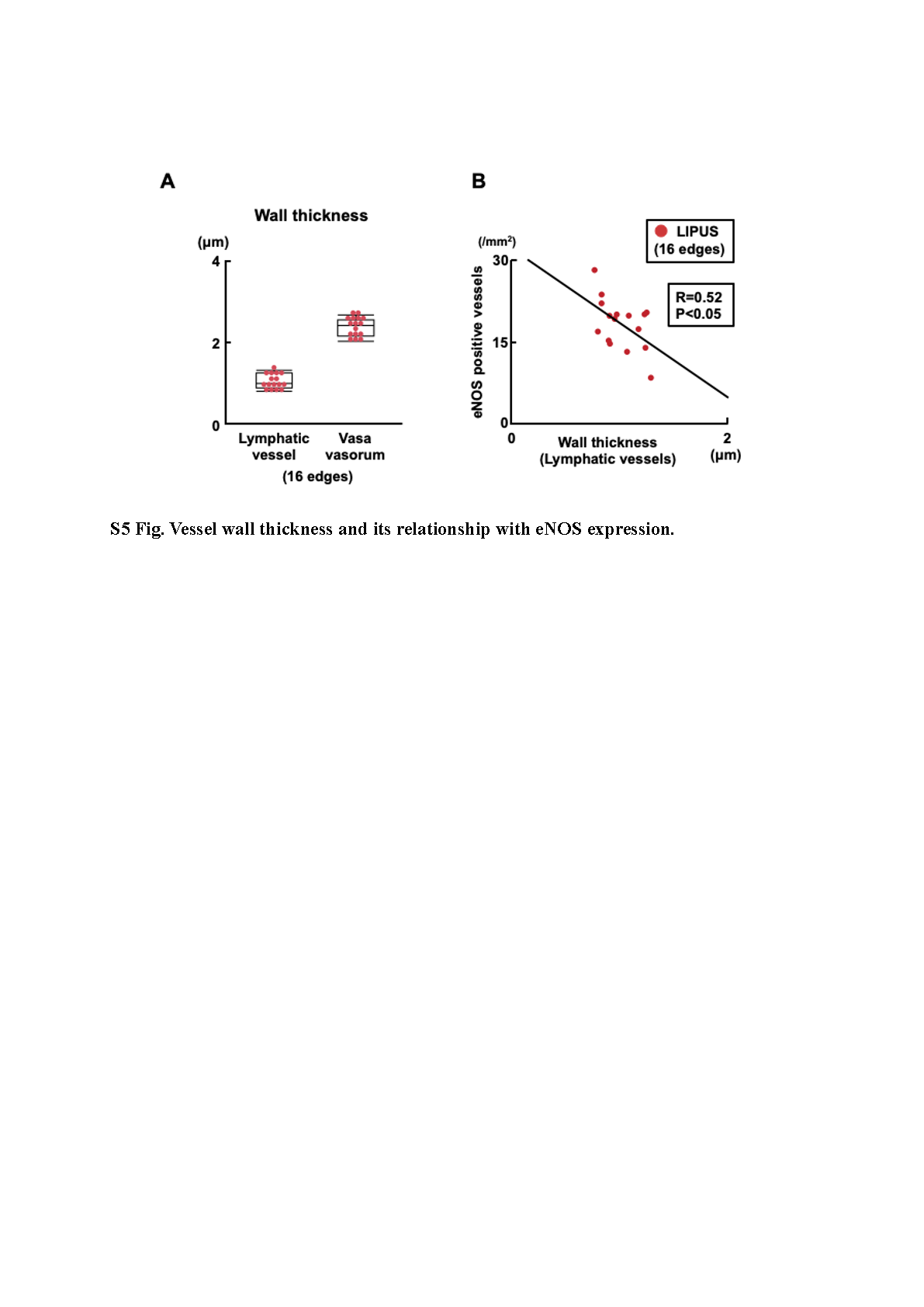

Supplement: S5 Fig — (A) Comparison of wall thickness of lymphatic vessels vs. vasa vasorum. (B) A significant positive correlation between lymphatic vessel wall thickness and eNOS positive vessels. Results are expressed as mean±SEM (A). Abbreviations as in S1 and S2 Figs. (TIFF) [file pone.0257175.s009.tiff]

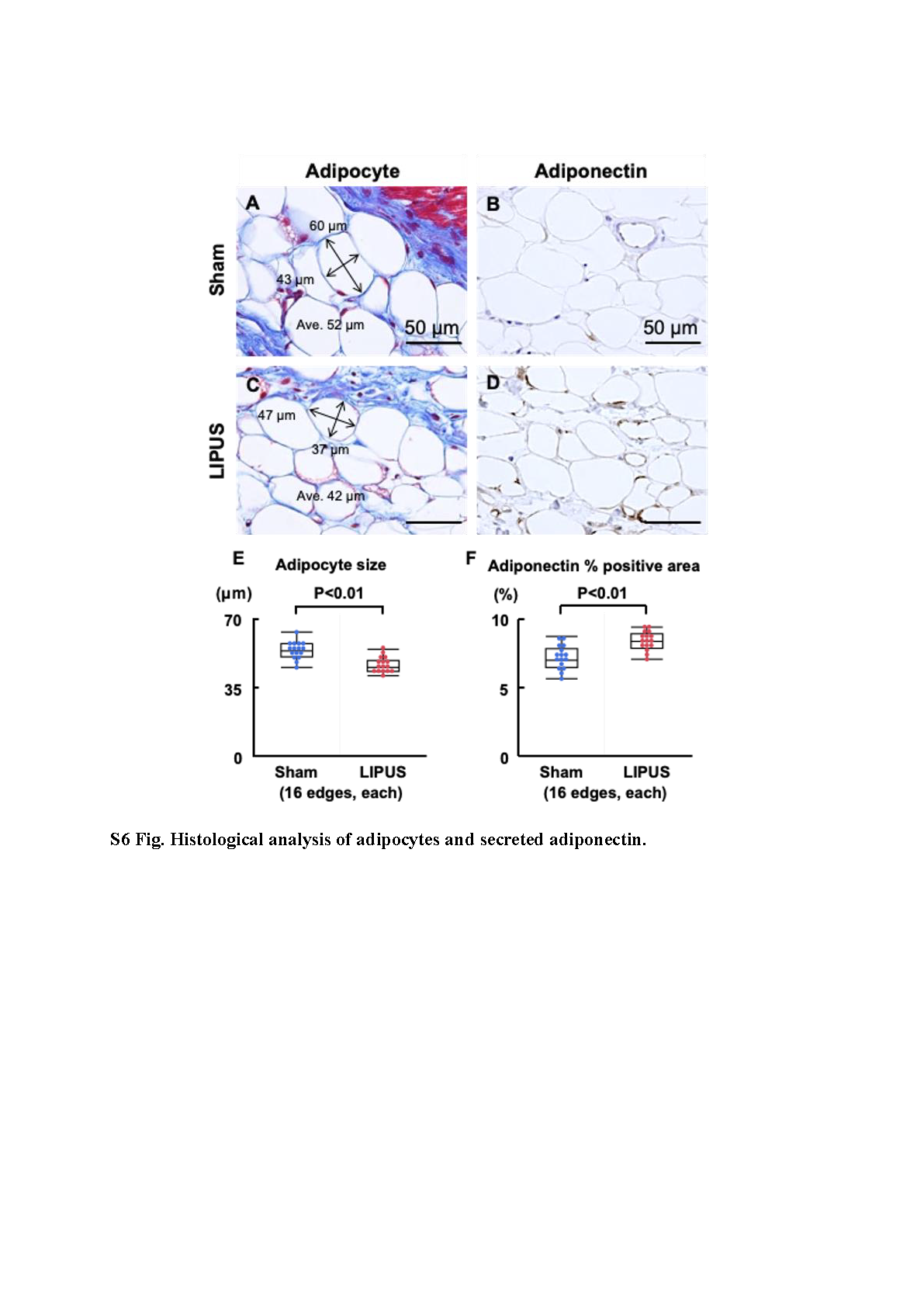

Supplement: S6 Fig — (A and C) MT stainings explaining the individual adipocyte measurement, and corresponding immunohistology of (B and D) adiponectin staining-positive cells of the perivascular adipose tissue of the vasoconstricting portions. Diameters in the perpendicular maximum and minimum axes (denoted by arrows) were measured (A and C). (E) Adipocyte was significantly smaller in the LIPUS group as compared with the sham group. (F) Adiponectin % positive area was significantly greater in the LIPUS group as compared with the sham group. Results are expressed as mean±SEM. Abbreviations as in S1 and S2 Figs. (TIFF) [file pone.0257175.s010.tiff]
