# Supplementary material for: Low-intensity pulsed ultrasound therapy suppresses coronary adventitial inflammatory changes and hyperconstricting responses after coronary stent implantation in pigs in vivo
Source: PLoS One. 2021 Sep 13;16(9):e0257175. doi: 10.1371/journal.pone.0257175 (PMC8437271; doi:10.1371/journal.pone.0257175)
Supplement: Attachment — Submitted filename: Re-1 Responses to Editor.docx [file pone.0257175.s012.docx]

Responses to the Editor

**Manuscript:** PONE-D-21-19896/R1

**Authors:** Watanabe T, et al.

**Title:** Low-intensity pulsed ultrasound therapy suppresses coronary adventitial inflammatory changes and hyperconstricting responses after coronary stent implantation in pigs in vivo

General comments

1. After careful consideration, we feel that it has merit but does not fully meet PLOS ONE's publication criteria as it currently stands. Therefore, we invite you to submit a revised version of the manuscript that addresses the points raised during the review process.

(Response)

We greatly appreciate the Editor for the favorable comments on our work. In line with the Reviewers’ comments, we have revised our manuscript. In order to facilitate the review process, our point-to-point responses are shown in a red font.

Specific comments

1. Only male pigs were used and the effect of LIPUS on female is not shown. If available, show female data. If not, discuss its limitation.

(Response)

Thank you very much for this important comment. Female hormone is known to have a vasculoprotective effect (Kawana A, et al. *Circ J* 2013;77:1267-1274.). In order to avoid the hormonal effect on coronary vasomotion, we used male pigs after castration. In the revised manuscript, we have quoted Kawana’s paper and have discussed this issue as one of the study limitations as follows;

(Methods section) (Page 3, para. 2, line 1)

Sixteen male Yorkshire pigs (3-4 month-old, male neutered, weighing 35-45 kg) were ---

(Discussion section) (Page 13, para. 2, lines 13-15)

Sixth, in order to avoid the effects of female hormones on coronary vasomotion [33], we used male pigs after castration. Thus, the present findings remain to be confirmed in female pigs.

(Reference)

33. Kawana A, Takahashi J, Takagi Y, Yasuda S, Sakata Y, Tsunoda R, et al. Gender differences in the clinical characteristics and outcomes of patients with vasospastic angina--a report from the Japanese Coronary Spasm Association. Circ J. 2013;77:1267-1274. <http://doi.org/10.1253/circj.cj-12-1486>. PMID: [23363662](https://pubmed.ncbi.nlm.nih.gov/23363662/)

2. Only everolimus-eluting stent was used, but the authors use the general term "DES" in the main text. Provide rationale to use everolimus-eluting stent. Are there any differences in the effects of LIPUS therapy on stent edge vasoconstriction and eNOS activity among DESs and other types of stent?

(Response)

Thank you very much for this important comment. We used an evelolimus-eluting stent (EES) for the following reasons. First, EES has been a most widely used DES in the real world. Second, we have previously demonstrated that due to inflammatory reaction to residual polymers, a DES with durable polymers enhances coronary vasoconstrictive responses more extensively as compared with a newer generation of DES with bioabsorbable polymer coating in pigs (Nishimiya K, et al. *Circ J* 2015;79:1787-1798*J Am Coll Cardiol* *Intv* 2016;9:281-291.). Third, we have recently reported that the number and caliber of lymphatic vessels were increased at the EES stent edges in pigs (Amamizu H, et al. *Arterioscler Thromb Vasc Biol* 2019;39:741-53.). Although we did not directly compare the effects of LIPUS therapy on stent edge coronary vasoconstriction or eNOS activity between the 2 types of DES, bioabosorbable polymer DES may exert different responses to the LIPUS therapy. In the revised manuscript, we have mentioned this point as follows;

(Discussion section) (Page 13, para. 2, lines 15-18)

Seventh, the effects of the LIPUS therapy on atherosclerotic lesions remain to be examined in future studies. We have recently reported that the number and caliber of lymphatic vessels were increased at the EES stent edges in pigs [9]. The effects of LIPUS on atherosclerotic lesions or a DES with biodegradable polymers [5] remain unknown.

3. Check the first page of references section.

(Response) We have formatted the **References** section accordingly. Thank you.

Finally, we again would like to thank the Editor for the valuable comments, which have greatly helped us improve our manuscript. We sincerely hope that our revised manuscript is now acceptable for publication in the Journal.
